# Evolution of hematopoietic stem cell potential from preterm to term neonates

**DOI:** 10.1002/hem3.70294

**Published:** 2026-02-09

**Authors:** Pamela Quaranta, Luca Basso‐Ricci, Luca Seffin, Guido Pacini, Andrea Ronchi, Riccardo Crimi, Monica Fumagalli, Carlo Pietrasanta, Serena Scala

**Affiliations:** ^1^ San Raffaele Telethon Institute for Gene Therapy (SR‐TIGET), San Raffaele Scientific Institute Milan Italy; ^2^ Università Vita‐Salute San Raffaele Milan Italy; ^3^ Neonatal Intensive Care Unit Fondazione IRCCS Ca' Granda Ospedale Maggiore Policlinico Milan Italy; ^4^ Università Degli Studi Di Milano Milan Italy; ^5^ Department of Clinical Sciences and Community Health, Dipartimento di Eccellenza 2023‐2027 Università Degli Studi Di Milano Milan Italy

During fetal development, hematopoiesis takes place in different organs, and the hematopoietic stem/progenitor cell (HSPC) output is tightly regulated by extrinsic signals provided by the distinct niches.^1^, ^2^ Moreover, HSPC intrinsic properties might contribute to the activation of the distinct waves of hematopoietic production to support fetal development and the maintenance of a tolerogenic environment between the mother and the fetus.^3^, ^4^ The liver is the main hematopoietic organ up to 24 weeks of gestation and the hematopoiesis is mainly erythroid‐biased, to support erythrocyte production for efficient oxygen delivery.^5^ At this stage, HSPCs start migrating toward the bone marrow (BM), but the end of this migratory process has not been defined. We have recently shown that young infants (0–3 months of age) have a higher number of circulating HSPCs (cHSPCs) vs. older children, suggesting that the liver‐to‐BM trafficking might continue for months after birth.^6^

In this dynamic context, preterm birth has been associated with a lower count of mature immune cells,^7^ a phenomenon that might contribute to the higher susceptibility to septic events typical of this population.^8^ This incomplete differentiation of preterm HSPCs could be due to their intrinsic reduced functionality or due to the absence of appropriate humoral signals from the BM hematopoietic niche supporting the generation of immune cells. Despite the increasing interest in characterizing preterm hematopoiesis, the HSPC features in this setting remained elusive and mainly focused on CB samples.^9^, ^10^, ^11^ Here, we aimed to investigate the biological properties of cHSPCs from term and preterm neonates as well as to evaluate the differences between preterm neonates affected or not by neonatal sepsis.

To investigate the biological features of cHSPCs of term and pre‐term neonates, we performed phenotypic and functional characterization on peripheral blood (PB) samples collected from very preterm neonates (PRET, with gestational age, GA, <32 weeks) and term neonates (TERM, GA ≥ 37 weeks). Detailed subjects' characteristics are reported in Supporting Information S1: Table S1. PB samples were collected within the first 24 h of life in EDTA pre‐filled tubes and processed within 6 h from blood draw. Neonatal sepsis (late‐onset sepsis, LOS) was defined as positive blood culture concomitant with clinical deterioration and need for antibiotic therapy according to the physician's evaluation beyond 72 h of life. The study was approved by the local ethical committee.

PB samples were analyzed using the whole‐blood dissection (WBD) protocol,^12^ labeling samples with fluorescent antibodies (Supporting Information S1: Table S2). Absolute cell quantification was performed by adding precision count beads (Biolegend) to samples before the WBD procedure. The gating strategy for the HSPC subset identification is reported in Supporting Information S1: Figure S1A and the identification of immune populations reported in Supporting Information S1: Table S3. Further methodological details are provided in the Supporting Information.

For the colony‐forming cell (CFC) assay, total vital cells deriving from 100 µL of PB were cultured in MethoCult H4434 (STEM CELL Technologies) according to the manufacturer's procedure. After 14 days, CFU were counted and classified according to their morphology. For in vitro multi‐lineage differentiation assays, 500 PB lineage (Lin)‐CD34+ cells (bulk) or single hematopoietic stem cells (HSC, Lin‐CD34 + CD38‐CD90 + CD45RA−) and multi‐potent progenitors (MPP, Lin‐CD34 + CD38‐CD90 − CD45RA−) were sorted and seeded on non‐tissue culture‐treated plates pre‐coated with StemSpan Differentiation Coating Material (Stem Cell Technologies). Cells were cultured in SFEM II medium (Stem Cell Technologies) supplemented with human recombinant cytokines (Supporting Information S1: Table S4). After 3 weeks of culture, cells were labeled with anti‐human conjugated antibodies (Supporting Information S1: Table S5). This assay was performed for all the subjects sampled with enough PB.

To investigate the differentiation dynamics of cHSPCs of term and pre‐term neonates, we evaluated their amount, phenotypic composition, and functional properties in 35 PRET (median GA: 28 weeks, median BW: 930 g) compared to 26 TERM (median GA: 39 weeks, median BW 3390 g), with a focus on primitive stem cells. To this end, we optimized a high‐throughput workflow for analyzing limited PB sample volumes by multi‐parametric immunophenotyping,^12^ as well as HSPC in vitro multi‐lineage differentiation^13^ and clonogenic potentials (Figure 1A).

**Figure 1:**
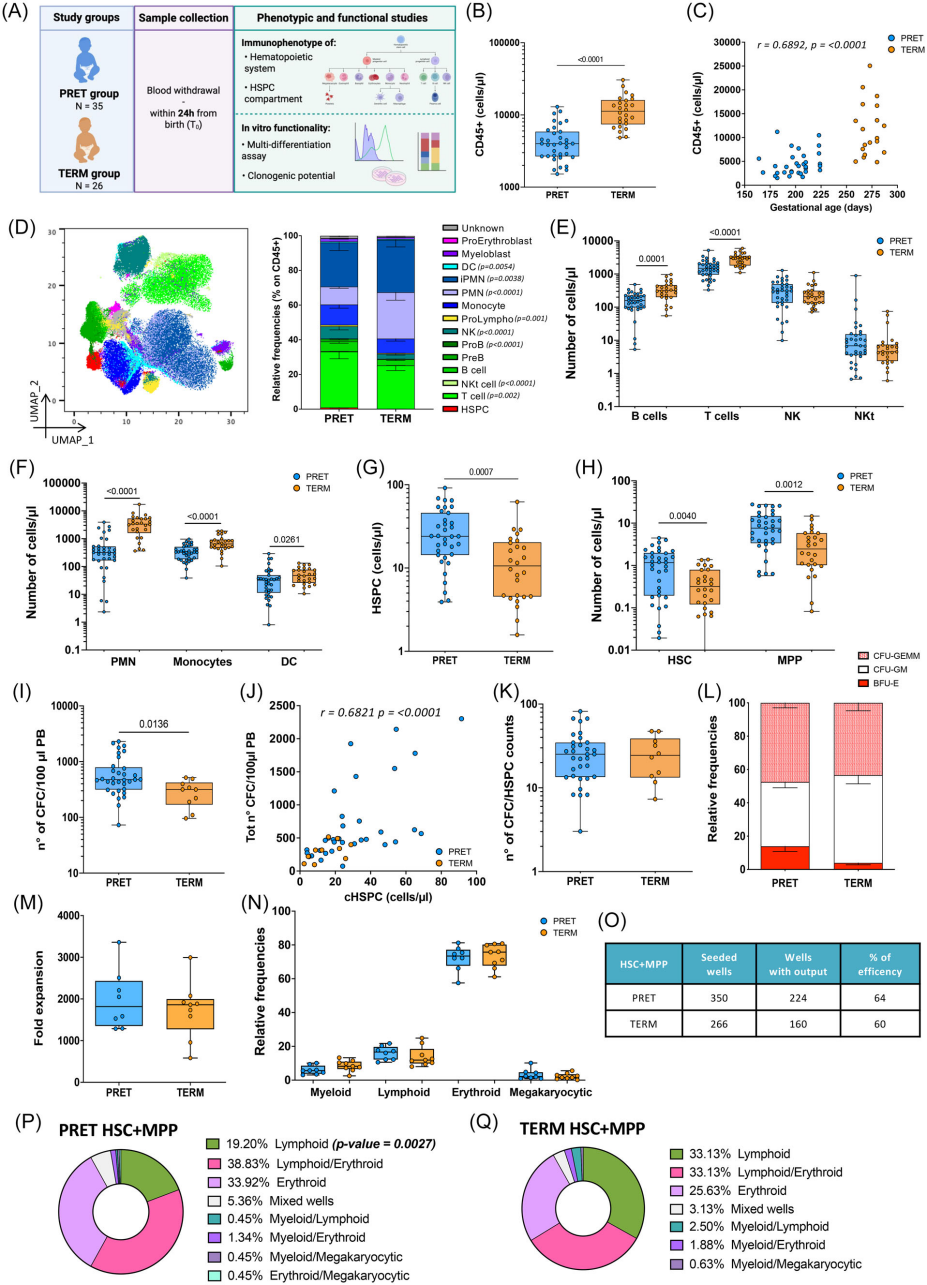
**PRET neonates show an immature hematopoietic system, with a functional skewing of primitive HSPCs toward erythroid differentiation**. **(A)** Sample workflow to characterize and study young infants' hematopoiesis. Gestational age (GA) cut‐off was set at 37 weeks to divide donors into preterm (PRET, *n* = 35) and term (TERM, *n* = 26) neonates. PB samples were used for immunophenotyping and HSPC‐dedicated functional studies. **(B)** CD45+ cell counts of PRET (blue) and TERM (orange) neonates. **(C)** Correlation between CD45+ cell counts and GA (days) at birth of both PRET and TERM children. Statistical test for correlation: Spearman *r*. Spearman's correlation coefficient (*r*) and p‐values are reported in the figure. **(D)** Uniform manifold approximation and projection (UMAP) embedding showing PB cell populations identified by multi‐parametric flow cytometry. Nonlinear dimensional reduction was applied to 100,000 randomly selected cells from the flow cytometry data set for 10 PRET and 10 TERM donors. The stacked bar graph shows the cell distribution in PRET and TERM groups. **(E, F)** Cell counts of lymphoid **(E)** and myeloid **(F)** cells in PRET and TERM groups. **(G)** Cell counts of Lin‐CD34+ HSPCs in PRET and TERM subjects. **(H)** Cell counts of primitive HSC and MPP in PRET and TERM groups. **(I)** Number of total CFC obtained from 100 µl of whole PB in PRET (blue) and TERM (orange) groups. **(J)** Correlation between the number of total CFC and the HSPC absolute count in PRET and TERM individuals. Statistical test for correlation: Spearman *r*. Spearman's correlation coefficient (*r*) and p‐value are reported. **(K)** CFC number normalized on the count of total HSPC in PRET and TERM. **(L)** Stacked bar graph showing the relative frequencies of BFU‐E, CFU‐GM, and CFU‐granulocyte, erythroid, macrophage, and megakaryocyte (CFU‐GEMM) generated from PRET and TERM PB samples. **(M)** Fold expansion of PRET and TERM HSPC at the end of the in vitro multi‐differentiation assay, calculated on the initial number of seeded cells. **(N)** Relative frequencies of myeloid, lymphoid, erythroid, and megakaryocytic differentiated cells derived from HSPC isolated from PRET (*n* = 8) and TERM (*n* = 9) subjects. **(O)** Summary of the differentiation efficiency of single HSC and MPP isolated from PRET (*n* = 7) and TERM (*n* = 6) in a single‐cell in vitro differentiation assay. The number of seeded wells, the count of wells showing differentiated progeny after 3 weeks of culture, and the total differentiation efficiency starting from one single cell are reported. **(P**, **Q)** Pie charts representing the LINAGE SCORES of PRET(P) and TERM (Q) HSC + MPP, based on the most abundant differentiation outputs toward lymphoid, myeloid, erythroid, and megakaryocyte lineages detected at the end of the single‐cell in vitro differentiation assay (see also Supporting Information Methods). **(B**, **E**–**I**, **K**, **M**, **N)** The Mann–Whitney statistical test was applied for groups' comparison, and single p‐values are reported within the graphs. Data are shown as median with interquartile range. HSPCs, hematopoietic stem/progenitor cells; MPP, multi‐potent progenitor; PB, peripheral blood.

PRET showed a reduced total PB CD45+ cell content with respect to TERM (Figure 1B), which positively correlated with subjects' gestational age and birth weight (Figure 1C and Supporting Information S1: Figure S1B). On comparing PRET and TERM hematopoietic composition, we detected a lower frequency of PMN and an increased relative frequency of the lymphoid compartment, including T, B, and Natural Killer (NK) cells in PRET (Figure 1D and Supporting Information S1: Figure S1C). On the other hand, we measured a lower cell count of mature lymphoid and myeloid PB populations as well as immature myelo/erythroid compartments in PRET (Figure 1E,F and Supporting Information S1: Figure S1D,E). It is noteworthy that PB from PRET was enriched for HSPCs, showing higher content of primitive hematopoietic stem cells (HSCs) and multi‐potent progenitors (MPPs) (Figure 1D,G,H and Supporting Information S1: Figure S1F–H). cHSPC number was inversely correlated with the GA (Supporting Information S1: Figure S1I). These data, consistent with previous analyses on CB^9^, indicate a lower degree of differentiation characterizing preterm hematopoietic system at birth. This may be attributed to (i) an intrinsically inefficient differentiation capacity of PRET‐HSPCs, leading to the accumulation of the most primitive HSPC subpopulations, and (ii) a consistent fraction of HSPCs migrating from the fetal liver (FL) to the BM at earlier gestational age,^14^ leading to a limited exposure to appropriate BM niche signals required for efficient hematopoiesis.

To investigate the intrinsic functional properties of PRET‐ and TERM‐HSPCs, we performed the CFC assay on 100 µL of PB. PRET‐HSPCs produced a higher number of colonies with respect to the TERM group, showing a positive correlation with subjects' HSPC count (Figure 1I,J). By normalizing the CFC count on the HSPC number, the differences between the two groups were abrogated, implying a comparable clonogenic potential of PRET‐ and TERM‐HSPCs under the same stimulating conditions (Figure 1K). PRET‐CFCs were enriched for burst‐forming unit‐erythroid (BFU‐E), while a higher production of granulocyte–monocyte colony‐forming unit (GM‐CFU) was observed in TERM samples (Figure 1L). To further characterize the hematopoietic output of PRET and TERM HSPCs, we applied an optimized multi‐lineage differentiation assay on PRET (*n* = 8)‐ and TERM (*n* = 9)‐derived Lin‐CD34+ cells and single sorted HSC + MPP from the two groups. We detected a comparable expansion rate and multi‐lineage hematopoietic output of bulk HSPCs, with a higher frequency of NK CD56+ cells in TERM than PRET progeny (Figure 1M,N and Supporting Information S1: Figure S2A,B). The single HSC + MPP assay showed similar differentiation efficiency of PRET and TERM HSC + MPP (Figure 1O), with PRET‐HSC + MPP displaying a 2.5‐fold higher expansion rate with respect to the TERM counterpart (Supporting Information S1: Figure S2C) due to a higher erythroid output, in line with CFU data (Supporting Information S1: Figure S2D and Figure 1L). On the other hand, TERM‐HSC + MPP showed increased relative lymphoid output (Supporting Information S1: Figure S2D). Consistently, when we scored each clone according to its uni‐, bi‐, and multi‐lineage output, we observed that PRET‐ and TERM‐HSC + MPP were, respectively, enriched in uni‐erythroid and uni‐lymphoid clones (Figure 1P,Q). Within the lymphoid compartment, PRET‐HSC + MPP produced more T cell precursors (T‐cell P) than the TERM counterpart, while a higher proportion of NK CD56+ cells was detected in TERM versus PRET lymphoid progeny, in line with bulk in vitro data (Supporting Information S1: Figure S2E,F).

Altogether, the comparable in vitro clonogenic and differentiation efficiency, along with the consistent multi‐lineage capability shown by PRET‐ and TERM‐HSPCs, implies similar intrinsic properties of HSPCs from distinct gestational ages. This suggests that the diverse mature hematopoietic output of HSPCs measured in the PB of PRET and TERM neonates at birth (Figure 1) might be the result of distinct extrinsic hematopoietic niche factors acting on HSPCs with the same potential, possibly reflecting the predominant role of the liver in early hematopoiesis and the progressive establishment of BM hematopoiesis at later stages.^15^, ^16^ Indeed, the enrichment of erythroid‐primed HSC + MPP observed in the PRET group is consistent with the erythroid skewing characterizing FL hematopoiesis, which serves as a primary source of red blood cells.^14^, ^17^ On the other hand, the BM micro‐environment providing Notch signaling,^18^ required for lymphoid differentiation, could explain the increased lymphoid output of HSC + MPP in TERM.

To further corroborate our interpretation, we analyzed the published scRNAseq data set of 1933 matched HSPCs from FL (*n* = 896) and BM (*n* = 1037) of human fetuses (17–22 weeks postconception).^19^ After integration, we identified 13 clusters associated with immature (HSC, MPP, erythroid‐MPP, myeloid/lymphoid‐MPP), myeloid (common myeloid progenitors‐granulocyte myeloid progenitors, CMP‐GMP; mono‐dendritic Progenitors, MDP), lymphoid (multi‐lymphoid progenitors, MLP; PreB and PreNK), megakaryocytic (Mkp), and erythroid (megakaryocyte erythrocyte progenitors, MEP; immature and mature erythroid progenitors) transcriptional programs (Supporting Information S1: Figure S2G). We found that HSPCs residing in the FL display increased frequency of erythroid clusters, while BM HSPCs show increased fraction of lymphoid clusters (Supporting Information S1: Figure S2H). Differential gene expression analysis of primitive cells from the two sources unveiled higher expression of genes involved in erythropoiesis in FL HSC + MPP (Supporting Information S1: Figure S2I), consistent with the in vitro erythroid skewing of PRET HSC + MPP (Figure 1P,Q and Supporting Information S1: Figure S2D). Altogether, these data suggest that the differentiation propensities of cHSC in TERM and PRET subjects reflect their tissue of origin: FL‐derived cells in PRET are skewed toward erythropoiesis, whereas BM‐derived cells in TERM display a lymphoid bias.

Given the higher risk of development of LOS in preterm neonates,^20^ we investigated whether distinct sepsis‐predicting signatures already exist at birth. To this end, we retrospectively identified in our PRET cohort subjects experiencing (S‐PRET, *n* = 17) or not (NS‐PRET, *n* = 16) LOS. For 2 PRET, the blood cultures were not available; thus, they were not included in this stratification. We performed multivariate logistic regression analysis, including both clinical variables and hematopoietic cell counts measured through our workflow, which highlighted that PRET neonates with lower GA as well as reduced CD45+ and DC cell counts were more prone to developing sepsis at later time points. Indeed, S‐PRET had lower counts of most mature lymphoid and myeloid populations, including DC, at birth, but not of HSPCs compared to NS‐PRET (Figure 2A–D and Supporting Information S1: Figure S3A–D). Moreover, for each hematopoietic population, we fitted a multivariate linear model to account for the effect of other clinical variables on the differences in the cell count observed between S‐PRET and NS‐PRET. This analysis revealed statistically significant differences in CD45+ (*P* = 0.045), monocyte (*P* = 0.042), and CD4 T cell (*P* = 0.049) counts between the two groups, while correcting for the other clinical parameters. These analyses suggest that within the PRET group, S‐PRET displayed an even higher level of immune system immaturity at birth, possibly predisposing to LOS. Finally, to infer the potential effect of the septic event on hematopoietic maturation, we longitudinally measured the hematopoietic profile of a subgroup of S‐PRET (*n* = 8) at birth (T0), at LOS onset (T1), and at sepsis resolution (14 days after T1, T2), with matched time points for the NS‐PRET (*n* = 11) counterpart (Figure 2E). We found that NS‐PRET have stable myeloid and HSPC compartment over time, with a trend toward an increase in T cell counts, in line with their adaptation to the external environment. On the other hand, S‐PRET showed an increased cell count of total CD45+ cells driven by a statistically significant increase of CMP, myeloblasts, and monocytes during the septic events, with partial normalization of the overall myeloid compartment after resolution (Figure 2F–M and Supporting Information S1: Figure S3E–J). These data imply that sepsis might affect hematopoiesis for a significant time after the resolution of the acute event.

**Figure 2:**
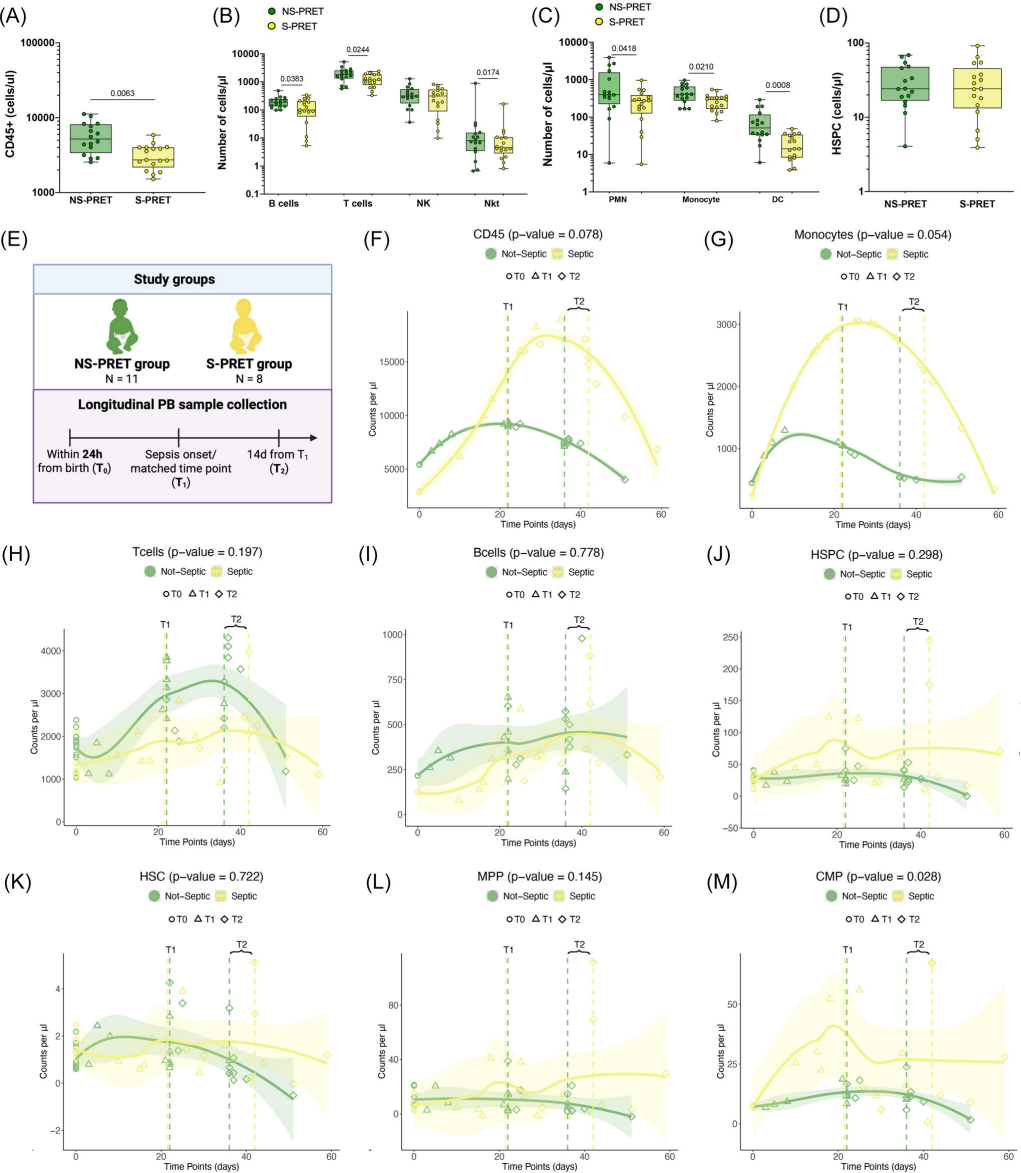
**Comparison of the hematopoietic compartment in septic and nonseptic PRET neonates**. **(A**–**D)** Cell counts of CD45+ cells **(A)**, mature lymphoid cells **(B)**, mature myeloid cells **(C)**, and HSPCs **(D)** in nonseptic (NS‐PRET) and septic (S‐PRET) preterm neonates. The Mann–Whitney statistical test was applied for groups' comparison and single p‐values are reported within the graphs. Data are shown as median with interquartile range. **(E)** Schematic representation of the workflow applied for analyzing longitudinally collected PB samples from NS‐PRET (*n* = 11) and S‐PRET (*n* = 8) groups. Samples were collected at birth (T0), at sepsis onset (T1), and after 14 days from T1 (T2), with matched time points for the NS‐PRET counterpart. **(F**–**M)** Number of total CD45+ cells **(F)**, monocytes **(G)**, T cells **(H)**, B cells **(I)**, HSPCs **(J)**, HSC **(K)**, MPP **(L)**, and CMP **(M)** detected over time (days) in the PB of NS‐PRET and S‐PRET groups. Predicted values (solid line) result from linear mixed‐effect models fit adopting cubic natural splines effects on time‐ and subject‐specific random slopes. Dashed vertical lines represent the median time point for NS‐ and S‐PRET samples collected at T1 and T2, while the shaded area represents the confidence interval of the predicted values. The p‐values referring to the difference in the trends over time between NS‐ and S‐PRET groups are reported on top. CMP, common myeloid progenitors; HSPCs, hematopoietic stem/progenitor cells; MPP, multi‐potent progenitor; PB, peripheral blood.

In summary, the functional properties of neonatal HSPCs evolve with the gestational age, from a more erythroid‐biased fate at earlier stages toward a more myeloid/lymphoid‐skewed composition due to distinct hematopoietic niche factors acting on HSPCs with similar differentiation properties. Moreover, our data support the hypothesis that a higher degree of immaturity of the hematopoietic system might increase the susceptibility to development of septic events in preterm neonates.

## AUTHOR CONTRIBUTIONS

**Pamela Quaranta**: Investigation; writing—original draft; methodology; formal analysis; visualization. **Luca Basso‐Ricci**: Investigation; writing—original draft; methodology; formal analysis; visualization. **Luca Seffin**: Investigation; writing—original draft; methodology; formal analysis. **Guido Pacini**: Data curation; formal analysis; visualization. **Andrea Ronchi**: Investigation; writing—original draft; formal analysis. **Riccardo Crimi**: Investigation; writing—original draft; formal analysis. **Monica Fumagalli**: Writing—review and editing; supervision. **Carlo Pietrasanta**: Conceptualization; investigation; writing—review and editing; supervision; data curation. **Serena Scala**: Conceptualization; investigation; writing—review and editing; supervision; funding acquisition; validation; data curation.

## CONFLICT OF INTEREST STATEMENT

The authors declare no conflict of interest.

## ETHICS STATEMENT

The study was approved by the local ethical committee (Milano area 2, register number: 986/2018), and written informed consent was obtained from the parents of enrolled neonates before any study‐related procedure.

## FUNDING

This work was supported by Fondazione Telethon (TGT21016 to S.S.) and the Italian Ministero della Salute (grant GR‐2019‐12369499 to S.S.). This study was (partially) funded by the Italian Ministry of Health—Current Research IRCCS. L.S. conducted this study as partial fulfillment of his PhD in Molecular Medicine, Gene and Cell Therapy program, San Raffaele University, Milan, Italy.

## Supporting information

Supporting Information.

## Data Availability

All the data generated in this study have been deposited in the San Raffaele Open Research Data Repository doi:10.17632/hvfphwt7xt.1.
