# Supplementary material for: Evolution of hematopoietic stem cell potential from preterm to term neonates
Source: Hemasphere. 2026 Feb 9;10(2):e70294. doi: 10.1002/hem3.70294 (PMC12885120; doi:10.1002/hem3.70294)
Supplement: Supplementary file 1 — Supporting Information. [file HEM3-10-e70294-s001.pdf]

Supplemental Methods

Flow cytometry data acquisition and analyses. All stained samples (from WBD or in vitro differentiation assay) were acquired through BD FACS Symphony A5 (BD Biosciences) cytofluorimeter after Rainbow bead (Spherotech) calibration. Raw FACS data were collected and analyzed through DIVA (BD Biosciences) and FlowJo (v10.5.3) software, respectively. The graphical output was generated through Prism v10.0.0 (GraphPad). Technically validated results were always included in the analyses, and we did not apply any exclusion criteria for outliers.

Lineage score for single-cell in vitro multi-lineage assay. For LINEAGE SCORE analysis, each single cell output was scored as uni-, bi- and multi-lineage based on the most abundant differentiation output toward lymphoid, myeloid, erythroid and megakaryocyte lineages. Uni-lineage clones were defined when >75% of produced cells belonged to only one lineage, while bi-lineage clones were characterized by two major differentiation outputs (each more than 20%) accounting for >70% of total cell output. Cells with heterogeneous output, not falling in the other two categories, were defined as mixed clones. The threshold for positive wells was determined based on negative controls.

Bioinformatic analyses of published scRNAseq data. HSPC from matched fetal liver (FL) and bone marrow (BM) samples of human fetuses were retrieved from a published scRNAseq dataset (E-MTAB-9067).[1] This dataset was analyzed using the Seurat[2], R package (v.4.3.0). The resulting count matrix was log-normalized (*NormalizeData*) and the 2000 most variable genes were identified (*FindVariableFeatures*). The gene-wise expression levels were scaled (*ScaleData*) accounting for the overall number of cell transcripts, the percentage of mtDNA transcripts and inferred cell cycle score (*CellCycleScoring*). Data integration (*RunHarmony*) was performed using the Harmony[3], R package (v.1.0), accounting for both the donor and HSPC source effects. Cells were then projected onto UMAP[4], using the top 77 harmonized PCs (*RunUMAP*). Clusters were identified using the Louvain algorithm through the *FindNeighbors* and *FindClusters* (*res = 1*) functions in Seurat, and downstream analyses were further restricted to the clusters with >50% of the cells expressing (count > 0) both *CD34* and *PTPRC* genes. The above normalization, scaling, dimensionality reduction and clustering procedures were repeated for this subset of cells. The resulting cell clusters (res = 1.9) were then annotated leveraging multiple independent reference annotation datasets. Differentially expressed genes between FL and BM derived HSC, MPP and HSC+MPP were then identified performing gene-

wise Wilcoxon's rank-sum tests (*FindMarkers*) and adjusting the nominal p-values by the Benjamini–Hochberg procedure to account for the multiple hypotheses testing issue.

Statistical model for sepsis prediction and longitudinal data analyses. To identify early predictors for sepsis in pre-term neonates, we performed penalized logistic regression using the Least Absolute Shrinkage and Selection Operator (LASSO) procedure[5], which accounts for correlated clinical variables and immunophenotypic cell count measurements performing coefficient shrinkage and identifying the most informative predictors. In this analysis the considered clinical variables were sex, gestational age, weight at birth, cause of preterm delivery and antenatal steroids. To account for the effects of clinical variables on the differences in the hematopoietic cell counts measured in S-PRET and NS-PRET subjects separately on each immunophenotypic population, we fitted a Generalized Linear Model (GLM) including the status (septic/not-septic) as well as all the abovementioned clinical measures as independent variables. The significance of the predictors' estimated coefficients was then tested with a Student's T-test (H0: coef = 0). To identify differences in trends over time between hematopoietic cell counts measured in S-PRET and NS-PRET subjects, we fit a linear mixed effect model for each immunophenotypic population with cubic natural splines effects on time and donor-specific random slopes and intercepts, using the lmerTest (v.3.1-3) and splines (v.4.1.3) R packages. Significant differences over time between the two conditions were then investigated testing the interaction term between the natural splines effects on days and the status variable through likelihood ratio tests (LRTs).

REFERENCES

1. Ranzoni AM, et al. Integrative Single-Cell RNA-Seq and ATAC-Seq Analysis of Human Developmental Hematopoiesis. *Cell Stem Cell*. 28(3), 472-487 e477 (2021).
2. Stuart, T. et al. Comprehensive integration of single-cell data. Cell 177, 1888–1902.e21 (2019).
3. Korsunsky, I. et al. Fast, sensitive and accurate integration of single-cell data with Harmony. Nat. Methods 16, 1289–1296 (2019).
4. McInnes, L. et al. UMAP: uniform manifold approximation and projection. J. Open Source Softw. 3, 861 (2018).
5. Tibshirani, R. et al. Regression shrinkage and selection via the Lasso. Journal of the Royal Statistical Society: Series B (Methodological), 58(1), 267–288 (1996).

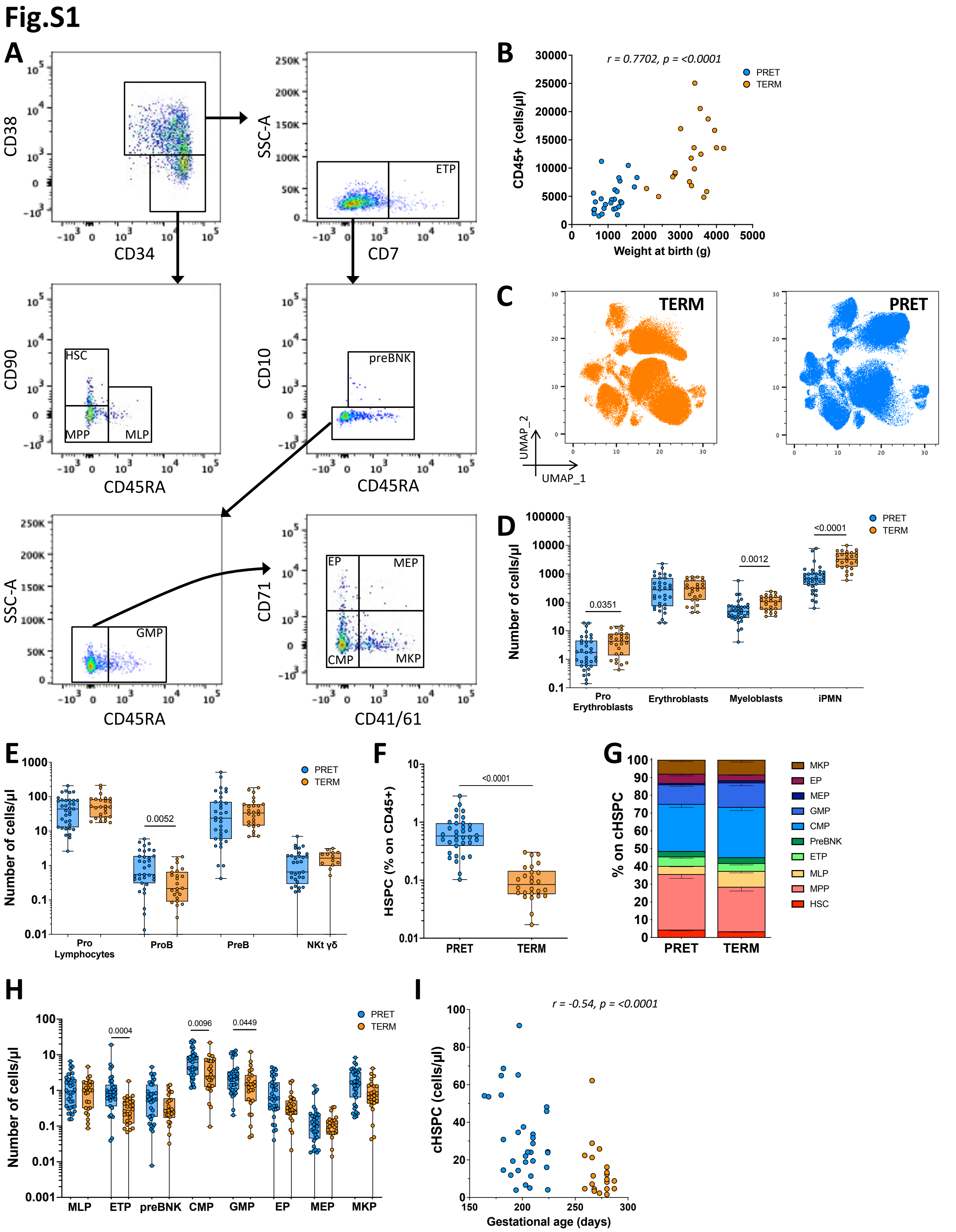

Fig.S1
A
CD38
CD34
SSC-A
CD7
ETP
CD90
CD45RA
HSC
MPP
MLP
CD10
preBNK
SSC-A
GMP
CD71
CD41/61
EP
MEP
CMP
MKP
B
r = 0.7702, p = <0.0001
PRET
TERM
CD45+ (cells/µl)
Weight at birth (g)
C
TERM
PRET
UMAP_2
UMAP_1
D
Number of cells/µl
0.0351
0.0012
<0.0001
Pro Erythroblasts
Erythroblasts
Myeloblasts
iPMN
E
Number of cells/µl
0.0052
Pro Lymphocytes
ProB
PreB
NKt γδ
F
HSPC (% on CD45+)
<0.0001
PRET
TERM
G
% on cHSPC
MKP
EP
MEP
GMP
CMP
PreBNK
ETP
MLP
MPP
HSC
H
Number of cells/µl
0.0004
0.0096
0.0449
MLP
ETP
preBNK
CMP
GMP
EP
MEP
MKP
I
r = -0.54, p = <0.0001
cHSPC (cells/µl)
Gestational age (days)

Figure S1. Immunophenotypic characterization of mature and immature peripheral blood cell populations in PRET and TERM groups. (A) Gating strategy for the identification of hematopoietic stem cells (HSC), multipotent progenitors (MPP), multi-lymphoid progenitors (MLP), early-T progenitors (ETP), precursors of B and NK cells (preBNK), granulocyte-monocyte progenitors (GMP), common myeloid progenitors (CMP), megakaryocyte-erythroid progenitors (MEP), erythroid progenitors (EP), megakaryocyte progenitors (MKP). **(B)** Correlation between CD45+ cell count and weight at birth of both PRET (blue) and TERM (orange) children. Statistical test for correlation: Spearman r. Spearman's correlation coefficient (r) and p-value are reported in the figure. **(C)** UMAP embeddings stratifying cells by PRET and TERM cells. **(D-E)** Cell counts of myelo/erythroid (D) and lymphoid (E) mature and immature cells in PRET and TERM individuals. **(F)** Relative frequencies of HSPC on CD45+ cells in PRET and TERM groups. **(G)** Stacked graph reporting relative frequencies of HSPC subpopulations in PRET and TERM donors. **(H)** Cell counts of lymphoid- (MLP, ETP, PreBNK), myeloid- (CMP, GMP) and erythroid/megakaryocytic- (EP, MEP, MKP) committed HSPC progenitors in PRET and TERM groups. **(I)** Correlation between cHSPC count and gestational age of both PRET (blue) and TERM (orange) children. Statistical test for correlation: Spearman r. Spearman's correlation coefficient (r) and p-value are reported in the figure. (D-F, H) Mann-Whitney statistical test was applied for groups' comparison, and single p-values were reported within the graphs. Data are shown as median with interquartile range.

Fig.S2

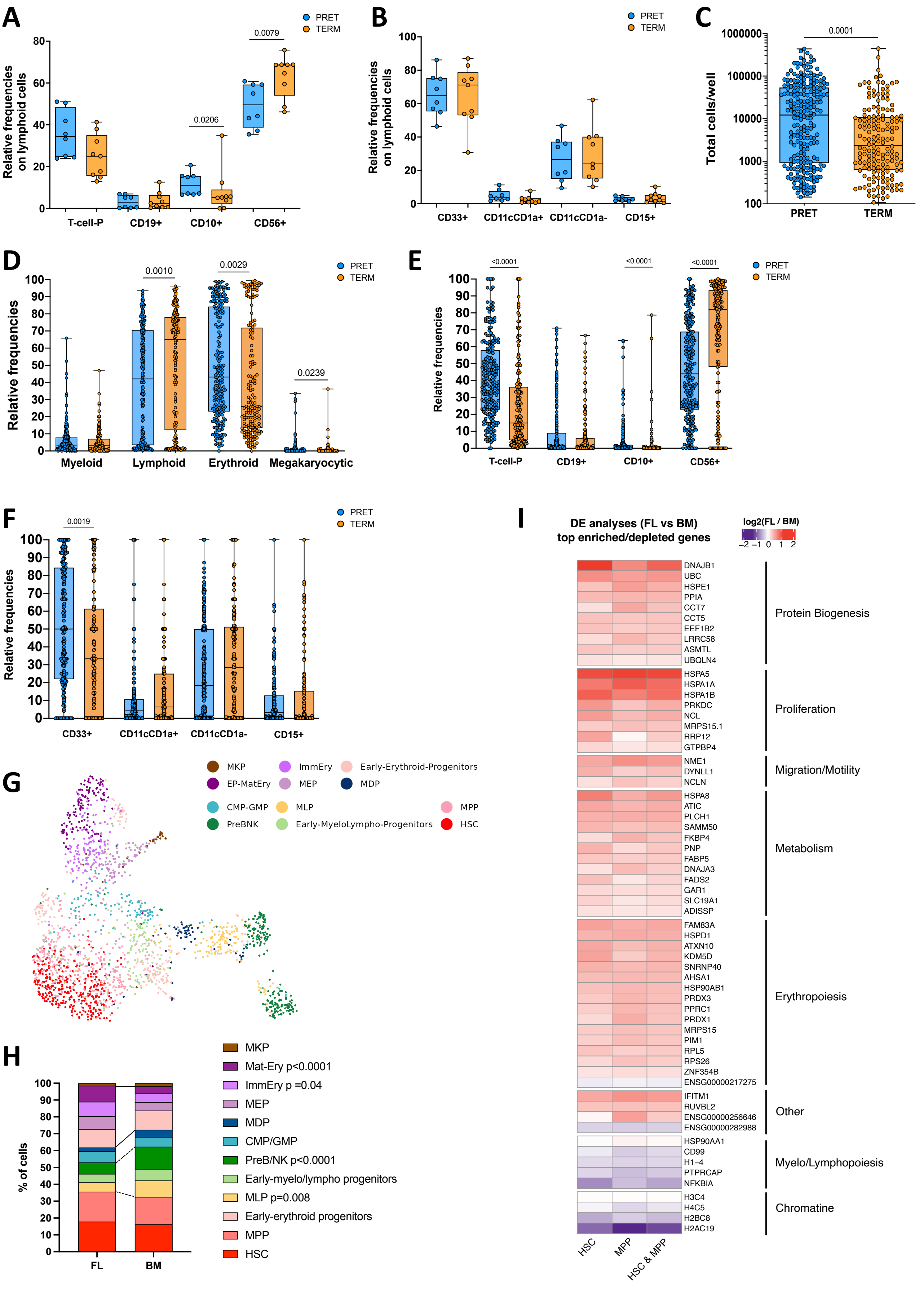

A
B
C
D
E
F
G
H
I
PRET
TERM
Relative frequencies on lymphoid cells
T-cell-P
CD19+
CD10+
CD56+
0.0206
0.0079
CD33+
CD11cCD1a+
CD11cCD1a-
CD15+
Total cells/well
0.0001
Relative frequencies
Myeloid
Lymphoid
Erythroid
Megakaryocytic
0.0010
0.0029
0.0239
<0.0001
0.0019
MKP
ImmEry
Early-Erythroid-Progenitors
EP-MatEry
MEP
MDP
CMP-GMP
MLP
MPP
PreBNK
Early-MyeloLympho-Progenitors
HSC
% of cells
FL
BM
Mat-Ery p<0.0001
ImmEry p =0.04
CMP/GMP
PreB/NK p<0.0001
Early-myelo/lympho progenitors
MLP p=0.008
Early-erythroid progenitors
DE analyses (FL vs BM)
top enriched/depleted genes
log2(FL / BM)
DNAJB1
UBC
HSPE1
PPIA
CCT7
CCT5
EEF1B2
LRRC58
ASMTL
UBQLN4
Protein Biogenesis
HSPA5
HSPA1A
HSPA1B
PRKDC
NCL
MRPS15.1
RRP12
GTPBP4
Proliferation
NME1
DYNLL1
NCLN
Migration/Motility
HSPA8
ATIC
PLCH1
SAMM50
FKBP4
PNP
FABP5
DNAJA3
FADS2
GAR1
SLC19A1
ADISSP
Metabolism
FAM83A
HSPD1
ATXN10
KDM5D
SNRNP40
AHSA1
HSP90AB1
PRDX3
PPRC1
PRDX1
MRPS15
PIM1
RPL5
RPS26
ZNF354B
ENSG00000217275
Erythropoiesis
IFITM1
RUVBL2
ENSG00000256646
ENSG00000282988
Other
HSP90AA1
CD99
H1−4
PTPRCAP
NFKBIA
Myelo/Lymphopoiesis
H3C4
H4C5
H2BC8
H2AC19
Chromatine
HSC
MPP
HSC & MPP

Figure S2. Functional characterization of PRET- and TERM-derived HSPC. (A-B) Relative frequencies of lymphoid (A) and myeloid (B) differentiated cells derived from 500 Lin-CD34+ HSPC isolated from PRET (n=8) and TERM (n=9) donors. Frequencies were calculated respectively on the number of differentiated lymphoid (A) and myeloid (B) cells, collected at the end of the experiment. Mann-Whitney statistical test was applied for groups' comparison, and single p-values were reported within the graphs. Data are shown as median with interquartile range. **(C)** Total cell count retrieved in each well at the end of the single-cell differentiation assay of primitive HSC and MPP derived from PRET (n=7) and TERM (n=7) groups. **(D)** Relative frequencies of myeloid, lymphoid, erythroid, and megakaryocytic differentiated cells derived from single primitive HSC and MPP isolated from PRET and TERM subjects. **(E-F)** Relative frequencies of lymphoid (E) and myeloid (F) differentiated cells derived from single HSC and MPP isolated from PRET and TERM donors. (C-F) Nonparametric two-tailed Student's t-test was performed to compare the single cells *in vitro* multi-differentiation outputs and single p-values were reported within the graphs. **(G)** UMAP embedding, showing the 13 Seraut clusters identified after unsupervised clustering of the fetal liver (FL) and bone marrow (BM)-matched HSPC scRNAseq dataset[21]. **(H)** Stacked bar graph showing the distribution of the transcriptional clusters in FL- and BM- HSPC. Fisher's exact tests, corrected with Bonferroni's multiple hypotheses testing adjustment procedure, were used to identify clusters with significantly (p-value < 0.05) different compositions between sources. **(I)** Heatmap showing the log2 fold-change values between fetal liver (FL) and bone marrow (BM) samples observed in HSC, MPP or HSC+MPP, for each significantly differentially expressed gene (Wilcoxon-Mann-Whitney test and BH adjustment procedure: p-value < 0.05). Up-regulated (n = 11, FL > BM) and down-regulated (n = 50, FL < BM) genes are grouped by biological function.

Fig.S3

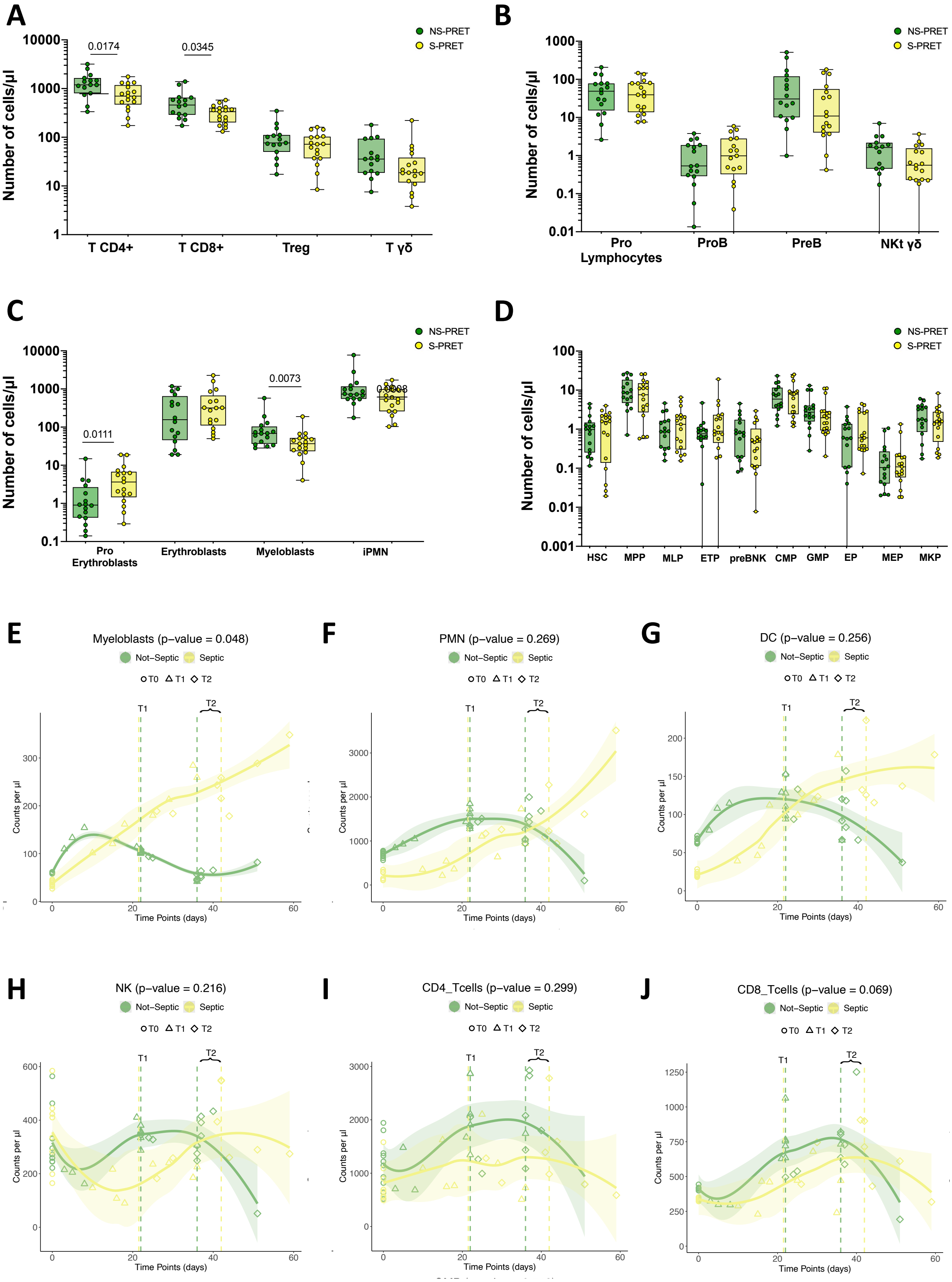
A
NS-PRET
S-PRET
Number of cells/µl
0.0174
0.0345
T CD4+
T CD8+
Treg
T γδ
B
Pro Lymphocytes
ProB
PreB
NKt γδ
C
0.0111
0.0073
Pro Erythroblasts
Erythroblasts
Myeloblasts
iPMN
D
HSC
MPP
MLP
ETP
preBNK
CMP
GMP
EP
MEP
MKP
E
Myeloblasts (p−value = 0.048)
F
PMN (p−value = 0.269)
G
DC (p−value = 0.256)
H
NK (p−value = 0.216)
I
CD4_Tcells (p−value = 0.299)
J
CD8_Tcells (p−value = 0.069)
Not−Septic
Septic
T0
T1
T2
Counts per µl
Time Points (days)

Figure S3. Comparison of the hematopoietic compartment in septic and non-septic PRET newborns. (A) Cell counts of mature T cell subsets in non-septic (NS-PRET) and septic (S-PRET) preterm children. **(B-C)** Cell counts of mature and immature lymphoid (B) and myelo/erythroid(C) cells in NS-PRET and S-PRET groups. **(D)** Cell counts of primitive, lymphoid, myeloid, erythroid/megakaryocytic HSPC subpopulations in NS-PRET and S-PRET groups. (A-D) Mann-Whitney statistical test was applied for groups' comparison and single p-values are reported within the graphs. Data are shown as median with interquartile range. **(E-J)** Number of myeloblasts (E), polymorphonuclear cells (PMN) (F), dendritic cells (G), natural killer cells (H) CD4 T cells (I) and CD8 T cells (J) detected overtime (days) in the PB of NS-PRET and S-PRET groups. Predicted values (solid line) result from linear mixed effect models fit adopting cubic natural splines effects on time and subject-specific random slopes. Dashed vertical lines represent the median time point for NS- and S-PRET samples collected at T1 and T2, while shaded area represent the confidence interval of the predicted values. The p-values referring to the difference in the overtime trends between NS- and S-PRET groups are reported on top.

Table S1. Clinical characteristics of term and preterm neonates enrolled in the study.

	Term born neonates (n=26)	Preterm born neonates (n=35)	
Females, n (%)	12 (46.1)	15 (42.8)	
Gestational age, weeks, median (range)	39 (37 - 41)	29 (23 - 32)	
Birth weight, grams, median (range)	3390 (2065 - 4200)	930 (570 - 2150)	
**Preterm born neonates (n=35)**	**without sepsis (n=16)**	**with sepsis (n=17)**	***p value***
Females, n (%)	6 (37.5)	8 (47.1)	*0,58*
Gestational age, weeks, median (range)	29 (23 - 32)	28 (24 - 32)	*0,042*
Birth weight, grams, median (range)	1220 (570 - 1800)	835 (615 - 2150)	*0,062*
PPROM, n (%)	10 (62.6)	6 (35.3)	*0,1*
IUGR, n (%)	3 (18.7)	5 (29.4)	*0,47*
Preeclampsia, n (%)	2 (12.5)	5 (29.4)	*0,23*
Antenatal steroids (at least 1 dose), n (%)	15 (93.7)	15 (88.2)	*0,783*
Age at sepsis onset, days, median (range)	-	15 (7 - 56)	*n.a.*
**Isolated pathogens**			
Coagulase-negative staphylococci		13 (76%)	*n.a.*
Staphylococcus aureus		1 (6%)	*n.a.*
Enterococci		1 (6%)	*n.a.*

*Calculated by Chi square or Mann-Whitney U test
PPROM: preterm-prelabor rupture of membranes
IUGR: intrauterine growth restriction

Table S2. List of fluorescent antibodies for the identification of hematopoietic populations

Antibody	Source	Identifier	Clone	Concentration
Mouse anti-human CD3 BV605	Biolegend	317322	OKT3	1:100
Mouse anti-human CD56 PC5	Biolegend	362516	5.1H11	1:100
Mouse anti-human CD14 BV510	Biolegend	301842	M5E2	1:100
Mouse anti-human CD33 BB515	BD Biosciences	564588	WM53	1:100
Mouse anti-human CD41/CD61 PC7	Biolegend	359812	A2A9/6	1:100
Mouse anti-human CD66b BB515	BD Biosciences	564679	G10F5	1:100
Mouse anti-human CD7 BB700	BD Biosciences	566488	M-T701	1:100
Mouse anti-human CD45 BUV395	BD Biosciences	563792	HI30	1:66
Mouse anti-human CD38 BUV737	BD Biosciences	612824	HB7	1:66
Mouse anti-human CD90 APC	BD Biosciences	559869	5E10	1:66
Mouse anti-human CD135 PE	Biolegend	313306	BV10A4H2	1:66
Mouse anti-human CD11c BV650	BD Biosciences	563404	B-ly6	1:20
Mouse anti-human CD10 BV786	BD Biosciences	564960	HI10a	1:20
Mouse anti-human CD34 BV421	Biolegend	343610	561	1:20
Mouse anti-human CD45RA APCH7	Biolegend	304128	HI100	1:20
Mouse anti-human CD71 BV711	BD Biosciences	563767	M-A712	1:20
Mouse anti-human CD19 APCR700	BD Biosciences	659121	SJ25C1	1:20

Table S3. Phenotypic markers to identify hematopoietic populations in PB samples

Hematopoietic population	Markers
iPMN	CD45+ CD33+ CD66b+ SShigh **CD10- and/or CD11c–**
PMN	CD45+ CD33+ CD66b+ **SShigh CD10+ CD11c+**
Monocyte	CD45+ CD33+ **CD14+**
DC	CD45+ CD33+ CD14- **CD11c+**
Myeloblast	CD45+ CD33+ CD14– CD11c– **CD34–**
T cells	CD45+ CD33– CD66b– **CD3+ CD56–**
NKt Cell	CD45+ CD33– CD66b– **CD3+ CD56+**
NK Cell	CD45+ CD33– CD66b– CD3- CD19- **CD56+**
B cell	CD45+ CD33– CD66b– CD3– **CD19+ CD10– CD34–**
Pre-B cell	CD45+ CD33– CD66b– CD3– **CD19+ CD10+ CD34–**
Pro-B cell	CD45+ CD33– CD66b– CD3– **CD19+ CD10+ CD34+**
Pro-lymphocyte	CD45+ CD33+ CD66b– CD3– CD19– CD56– CD34– CD71– CD41/61– **CD7+ or CD10+**
Pro-erythroblast	CD45+ CD33+ CD66b– CD3– CD19– CD56– CD34– **CD71+**
Erythroblast	**CD45- CD71+**
HSC	CD45+ CD14– CD11c– CD3– CD19– CD56– **CD34+ CD38– CD90+ CD45RA-**
MPP	CD45+ CD14– CD11c– CD3– CD19– CD56– **CD34+ CD38– CD90- CD45RA-**
MLP	CD45+ CD14– CD11c– CD3– CD19– CD56– **CD34+ CD38– CD90+ CD45RA+**
ETP	CD45+ CD14– CD11c– CD3– CD19– CD56– **CD34+ CD38+ CD7+**
PreBNK	CD45+ CD14– CD11c– CD3– CD19– CD56– **CD34+ CD38+ CD7- CD10+**
GMP	CD45+ CD14– CD11c– CD3– CD19– CD56– **CD34+ CD38+ CD7- CD10- CD45RA+**
CMP	CD45+ CD14– CD11c– CD3– CD19– CD56– **CD34+ CD38+ CD7- CD10- CD45RA- CD71- CD41/61-**
MEP	CD45+ CD14– CD11c– CD3– CD19– CD56– **CD34+ CD38+ CD7- CD10- CD45RA- CD71+ CD41/61+**
EP	CD45+ CD14– CD11c– CD3– CD19– CD56– **CD34+ CD38+ CD7- CD10- CD45RA- CD71+ CD41/61-**
MKP	CD45+ CD14– CD11c– CD3– CD19– CD56– **CD34+ CD38+ CD7- CD10- CD45RA- CD71- CD41/61+**

Table S4. List of cytokines for multi-lineage differentiation assay

Cytokine	Source	Identifier	Final concentration
Human stem cell factor (hSCF)	Peprotech	300-07	100 ng/ml
Human FLT3-L	Peprotech	300-19	10 ng/ml
Human thrombopoietin (hTPO)	Peprotech	300-18	75 ng/ml
Human Interleukin-3 (hIL-3)	Peprotech	200-03	10 ng/ml
Human Interleukin-7 (hIL-7)	Peprotech	200-07	100 ng/ml
Human Interleukin-2 (hIL-2)	Novartis	27131010	10 ng/ml
Human Interleukin-6 (hIL-6)	Peprotech	200-06	40 ng/ml
Human Interleukin-11 (hIL-11)	Peprotech	200-11	50 ng/ml
Human Erythropoietin (hEPO)	Peprotech	100-64	0.1 U/ml
Human Interleukin-4 (hIL-4)	Miltenyi Biotec	130-093-917	10 ng/ml
hLDL	Stem Cell technologies	2698	4 µg/ml

Table S5. List of antibodies for analyzing the hematopoietic output of HSPC upon multi-lineage differentiation assay

Antibody	Source	Identifier	Clone	Concentration
Mouse anti-human CD235a PE	BD Biosciences	561051	GA-R2 (HIR2)	1:200
Mouse anti-human CD1a APC	BD Biosciences	561755	HI149	1:200
Mouse anti-human CD5 BUV737	BD Biosciences	612842	UCHT2	1:66
Mouse anti-human CD42b BV786	BD Biosciences	740976	HIP1	1:20
Mouse anti-human CD41 PC7	Biolegend	303718	HIP8	1:100
Mouse anti-human CD10 BV510	Biolegend	312220	H10a	1:200
Mouse anti-human CD15 APC fire750	Biolegend	323041	W6D3	1:100
Mouse anti-human CD3 BV605	Biolegend	317322	OKT3	1:100
Mouse anti-human CD56 PC5	Biolegend	362516	5.1H11	1:100
Mouse anti-human CD33 BB515	BD Biosciences	564588	WM53	1:100
Mouse anti-human CD7 BB700	BD Biosciences	566488	M-T701	1:100
Mouse anti-human CD45 BUV395	BD Biosciences	563792	HI30	1:66
Mouse anti-human CD34 BV421	Biolegend	343610	561	1:20
Mouse anti-human CD11c BV650	BD Biosciences	563404	B-ly6	1:20
Mouse anti-human CD71 BV711	BD Biosciences	563767	M-A712	1:20
Mouse anti-human CD19 APCR700	BD Biosciences	659121	SJ25C1	1:20
